# Cell cycle and HIF-1 related gene expression alteration in thyroid cell lines under microgravity

**DOI:** 10.32604/or.2025.065847

**Published:** 2025-07-18

**Authors:** JONG-HYUK AHN, JIN WOOK YI

**Affiliations:** 1 Department of Surgery, Chung-Ang University Hospital, Seoul, 06974, Republic of Korea; 2 Department of Surgery, Inha University College of Medicine, Incheon, 22209, Republic of Korea; 3 Department of Surgery, Inha University Hospital, Incheon, 22332, Republic of Korea

**Keywords:** Weightlessness, Space simulation, Thyroid neoplasms, cDNA microarray, Gene expression profiling, Gene ontologies, Cell migration assays

## Abstract

**Background:**

With growing interest in space exploration, understanding microgravity’s impact on human health is essential. This study aims to investigate gene expression changes and migration and invasion potential in five thyroid-related cell lines cultured under simulated microgravity.

**Methods:**

Five thyroid-related cell lines—normal thyrocytes (Nthy-ori 3-1), papillary thyroid cancer (PTC) cells (SNU-790, TPC-1), poorly differentiated thyroid cancer cell (BCPAP), and anaplastic thyroid cancer cell (SNU-80)—were cultured under simulated microgravity (10^−3^ g) using a clinostat. Differentially expressed genes (DEGs) were analyzed using cDNA microarray, followed by functional annotation and assessment of aggressiveness via Transwell migration and invasion assays.

**Results:**

DEG analysis under simulated microgravity revealed distinct gene expression profiles by gravity condition, with 2980 DEGs in SNU-790, 1033 in BCPAP, 562 in TPC-1, 477 in Nthy-ori 3-1, and 246 in SNU-80, as confirmed by hierarchical clustering. In PTC cell lines (SNU-790, TPC-1), G2–M phase–related genes were upregulated. In non-PTC cell lines (BCPAP, SNU-80), genes associated with innate immune response, Toll-like receptor signaling, were upregulated, whereas Hypoxia-Inducible Factor 1-alpha (HIF-1α) signaling-related genes were downregulated. Additionally, under simulated microgravity, significant migration was observed in SNU-790 (3 × 10^4^ cells) and BCPAP (2 × 10^4^ and 3 × 10^4^), while significant invasion occurred in SNU-790, Nthy-ori 3-1, and BCPAP at a seeding density of 2 × 10^4^. Other conditions showed no significant differences.

**Conclusion:**

This study comprehensively evaluates the effects of simulated microgravity using a diverse panel of thyroid-related cell lines. These findings provide valuable insight into how microgravity could influence cancer biology, emphasizing the importance of further research on cancer behavior in space environments and its implications for human health during long-term space missions.

## Introduction

With the increased focus on space exploration and long-term habitation projects, such as Artemis, health issues in space have become a critical concern. These challenges are linked to exposure to unique conditions, such as altered gravity, vibrations, and radiation, which differ significantly from those on Earth [1]. Gravity in space is different from that on Earth, and the gravitational force approaches microgravity. Gravity is essential for maintaining homeostasis in tissues and cells during biological processes [2]. Consequently, the effects of microgravity on the human body have become a significant focus of ongoing research [3].

Microgravity differs significantly from the typical gravitational environment on Earth, offering new insights into fundamental cellular biological processes, such as cell differentiation, proliferation, growth, programmed cell death, adhesion, migration, and invasion [1,4,5]. In particular, understanding how characteristics, such as cancer cell growth, differentiation, migration, and invasion, change in microgravity can provide critical clues on the intrinsic nature of cancer and potential treatment strategies [6,7].

Thyroid cancer is prevalent worldwide, but the mechanisms underlying its development are unclear [8]. Moreover, no effective treatments are currently available for conditions like radioiodine-refractory thyroid cancer or anaplastic thyroid cancer (ATC), making the development of new therapeutic strategies a pressing issue. Astronauts and airline crew, due to continuous exposure to natural sources of radiation such as galactic cosmic rays, solar particle events, and the Earth’s radiation belts outside the shielding of the Earth’s atmosphere and magnetic field, are subject to whole-body radiation that may particularly affect radiosensitive organs like the thyroid gland [9]. Previous research has shown that ionizing radiation, a characteristic of space, is associated with a higher standardized incidence ratio (1.11) and standardized mortality ratio (1.19) for thyroid cancer in airline crew members [10]. A recent national cohort study in Korea found that female airline crew members had a significantly higher standardized incidence ratio (SIR) of 1.25 for thyroid cancer compared to the general population, suggesting a potential association with occupational factors such as cosmic radiation [11]. Hence, the space environment may have a greater impact on humans than the exposure experienced by airline crew members with regard to thyroid cancer. Furthermore, several studies have reported that microgravity induces morphological and biological changes in thyroid cancer cells [4,5,7,12,13]. In addition, changes in the immune system caused by microgravity, along with environmental and dietary changes in space, can produce conditions that adversely impact thyroid cancer [14].

Most previous studies on thyroid cancer in microgravity have focused on a limited number of Western-derived follicular thyroid cancer cell lines. Despite these findings, limited research has explored how microgravity affects the gene expression landscape and aggressive behaviors of various thyroid cancer cell types. To address this gap, we selected five thyroid-related cell lines that reflect a wide spectrum of histological differentiation and genetic diversity. This panel includes papillary thyroid carcinoma (PTC) cell lines, which represent the most prevalent subtype of thyroid cancer worldwide, as well as poorly differentiated and anaplastic thyroid carcinoma lines, which are associated with aggressive clinical behavior and limited treatment options. A normal thyrocyte line was also included as a physiological reference. This diversity allows a comprehensive comparison of microgravity effects across the spectrum of thyroid cancer progression and differentiation. Given the diversity of thyroid malignancies and their differing genetic backgrounds, a comparative evaluation under microgravity conditions may uncover novel insights into cancer progression in extraterrestrial environments.

We hypothesized that microgravity induces distinct gene expression changes and promotes the migratory and invasive behavior of thyroid cancer cells. To test this, we examined molecular and functional alterations in five thyroid-related cell lines cultured under simulated microgravity, including gene expression profiling and transwell migration and invasion assays.

## Materials and Methods

### Five thyroid cell lines

This study analyzed the characteristics of five thyroid cell lines (Nthy-ori 3-1, SNU-790, SNU-80, TPC-1, BCPAP) as listed in Table 1. The SNU-790 and SNU-80 cell lines were purchased from the Korea Cell Line Bank (Seoul, Korea, https://cellbank.snu.ac.kr, accessed on 22 May 2025), while the TPC-1, BCPAP, and Nthy-ori 3-1 cell lines were provided by the Seoul National University Cancer Research Institute (Seoul, Korea, https://cri.snu.ac.kr, accessed on 22 May 2025). All cell lines were authenticated using short tandem repeat profiling and tested negative for mycoplasma contamination prior to experiments. SNU-790 and TPC-1 were used as PTC cell lines; these two cell lines do not share common genetic mutations [15,16]. In contrast, Nthy-ori 3-1, SNU-80, and BCPAP are not associated with PTC [15,17,18]. Specifically, Nthy-ori 3-1 is a permanent normal thyroid follicular epithelial cell line, SNU-80 is an ATC cell line, and BCPAP is a poorly differentiated thyroid cancer (PDTC) cell line.

**Table 1:** Characteristics of the five thyroid cell lines used in the research

	Nthy-ori 3-1	SNU-790	SNU-80	TPC-1	BCPAP
Origin	35 yr female	72 yr Korean male	59 yr Korean female	Adult, Japanese female	76 yr Caucasian Female
Characteristics	Normal thyrocyte	Papillary thyroid cancer	Anaplastic thyroid cancer	Papillary thyroid cancer	Poorly differentiated thyroid cancer
Genetic mutation		*BRAF* (c.1799T > A)	*BRAF* (c.1405G > C)	*RET/PTC1*	*BRAF* (c.1799T > A)
				*CDKN2A* (c.204_208delGGAGC)	
					*TERT* (c.228C > T)
			*TP53* (c.832C > G)		
				*STAG2* (c.3265C > T)	*TP53* (c.775G > T)
				*TERT* (c.228C > T)	

The genetic mutation characteristics showed that the SNU-790 and BCPAP cell lines carry the *BRAF* (c.1799T > A) mutation, while the SNU-80 cell line has the *BRAF* (c.1405G>C) mutation. In addition, the *TERT* (c.228C > T) mutation was identified in the TPC-1 and BCPAP cell lines. The *TP53* (c.832C > G) mutation was observed in the SNU-80 cell line, whereas the *TP53* (c.775G > T) mutation was observed in the BCPAP cell line. Further details about these cell lines can be accessed through the Cellosaurus database (https://www.cellosaurus.org/, accessed on 22 May 2025).

### Cell culture under simulated ground-based microgravity and normal gravity

The cell culture and microgravity setup for this study followed a previously established protocol [19]. Five cell lines were seeded at 1 × 10^6^ cells into T-25 culture flasks (Corning, Oneonta, NY, USA) and incubated in a humidified environment with 5% CO_2_ at 37°C. The cells were cultured in RPMI-1640 medium (Cytiva, Marlborough, MA, USA) supplemented with 10% fetal bovine serum (FBS; Cytiva, Marlborough, MA, USA) and 1% penicillin-streptomycin (Thermo Fisher Scientific, Waltham, MA, USA).

After 24 h of subculture, each of the five thyroid-related cell lines was reseeded into six T-25 culture flasks (Corning, Corning, NY, USA) at a density of 1 × 10^6^ cells per flask with the media conditions remaining consistent with those used during the subculture. The six flasks for each cell line were then divided equally into two groups: three flasks for the simulated microgravity condition and three for the normal gravity control. Simulated microgravity was generated using clinostat equipment (Gravite®, Space Bio-Laboratories Co., Ltd., Hiroshima, Japan). The clinostat (Gravite®, Space Bio-Laboratories Co., Ltd., Hiroshima, Japan) simulates microgravity by continuously rotating the culture flask around a horizontal axis, thereby averaging the gravitational vector over time and effectively minimizing the net gravitational force experienced by the cells. This approach has been shown to effectively simulate microgravity conditions (10^–3^ g), yielding results comparable to those obtained under real microgravity in various biological systems, thus justifying its widespread use in ground-based studies [20]. The flasks assigned to the microgravity group were mounted on a rotating holder of the clinostat to simulate a gravitational force of 10^–3^ g microgravity. The control group flasks were placed stationary at the bottom of the same clinostat chamber to maintain standard Earth gravity. This setup was designed to ensure that gravity was the only differing variable, while all other environmental conditions were strictly controlled and consistent between the two groups. All flasks were placed inside the clinostat chamber and incubated for 120 h (5 days) without media replacement.

### cDNA microarray

The adherent cells from flasks cultured for five days under microgravity and normal gravity conditions were collected for RNA extraction using the guanidium-acid-phenol extraction method [21]. After washing with phosphate-buffered saline (1× PBS, pH 7.0–7.2, without calcium, magnesium, or phenol red; sterile-filtered through a 0.1 μm filter; Cytiva, Marlborough, MA, USA), cell lysis was induced using TransZol (TransGen Biotech Co., Ltd., Beijing, China) to inactivate endogenous ribonuclease (RNase) activity and selectively isolate RNA. Chloroform (Merck KGaA, Darmstadt, Germany) was added to separate the RNA from DNA and protein, and the resulting aqueous phase was collected. Isopropanol (Merck KGaA, Darmstadt, Germany) was added to precipitate the RNA. The RNA was pelleted by centrifugation at 13,000 g for 10 min at 4°C and washed with 80% ethanol (Thermo Fisher Scientific, Waltham, MA, USA) to remove metal salt ions. The RNA pellet was air-dried and dissolved in distilled water (Thermo Fisher Scientific, Waltham, MA, USA) to induce the alkylation of histidine at the RNase activation sites.

The RNA quality was evaluated thoroughly using a NanoDrop spectrophotometer (Thermo Fisher Scientific, Waltham, MA, USA). The RNA concentration was determined by measuring the absorbance at 260 nm, with purity ratios of 1.7–2.0, indicating relatively pure RNA. The RNA integrity was assessed, and a minimum RNA integrity number of eight was required.

The GeneChip Human Gene 2.0 ST Array (Thermo Fisher Scientific, Waltham, MA, USA) was utilized for cDNA microarray analysis. cDNA synthesis, fragmentation, and biotin labeling were conducted using the GeneChip WT Amplification Kit and the GeneChip WT Terminal Labeling Kit (Thermo Fisher Scientific, Waltham, MA, USA). After hybridizing the cDNA to the Affymetrix GeneChip Array (Affymetrix Inc., Santa Clara, CA, USA), the arrays were washed and stained on a GeneChip Fluidics Station 450 (Thermo Fisher Scientific, Waltham, MA, USA) and scanned with a GCS3000 Scanner (Affymetrix Inc., Santa Clara, CA, USA). The probe cell intensity data were calculated, and CEL files were generated using the Analysis Power Tools version 2.11.6 (Thermo Fisher Scientific, Waltham, MA, USA).

### Differentially expressed genes analysis and Functional Annotation analysis

Differentially Expressed Genes (DEGs) analyses were conducted using Transcriptome Analysis Console Software version 4.0 (Thermo Fisher Scientific, Waltham, MA, USA). DEGs between the microgravity and normal gravity conditions were analyzed. A fold change (FC) cutoff of 1.5 was used to determine the significant DEGs. Genes with a *p* < 0.05 or a false discovery rate (FDR) < 0.2 were considered significant. Hierarchical clustering analysis was performed to determine if the DEG patterns differed based on the gravity conditions. For DEGs that were commonly upregulated or downregulated across two or more cell types under microgravity, the analysis was carried out using Bioinformatics and Evolutionary Genomics tools (Gent, Belgium; https://bioinformatics.psb.ugent.be/webtools/Venn/, accessed on 22 May 2025).

Functional analysis of DEGs between the two gravity conditions was performed using several functional annotation databases, including the Gene Ontology (GO) (http://geneontology.org, accessed on 22 May 2025), Kyoto Encyclopedia of Genes and Genomes (KEGG) pathway (http://kegg.jp, accessed on 22 May 2025), and Gene Set Enrichment Analysis (GSEA) (https://www.gsea-msigdb.org, accessed on 22 May 2025). GO and KEGG analyses were conducted using g:Profiler (https://biit.cs.ut.ee/gprofiler/gost, accessed on 22 May 2025), while GSEA analysis was carried out using the software provided by the Broad Institute (https://www.gsea-msigdb.org/gsea/index.jsp, accessed on 22 May 2025). This study examined whether the GO terms, KEGG pathways, or GSEA gene sets with significant enrichment were common across two or more cell types.

### Transwell migration and invasion assay

Transwell migration and invasion assays were used to evaluate the aggressiveness of thyroid-related cells cultured under simulated microgravity conditions. The cells were grown in simulated microgravity and normal gravity for five days and seeded into the insert wells at three different densities: 1 × 10^4^, 2 × 10^4^, and 3 × 10^4^ cells per well. The insert wells were filled with serum-free media (Hyclone, Thermo Fisher Scientific, Waltham, MA, USA), including RPMI 1640 Medium supplemented with 25 mM 4-(2-Hydroxyethyl)piperazine-1-ethanesulfonic acid (HEPES) and 2 mM L-glutamine, while complete growth media was added to the lower chamber to act as a chemoattractant. After incubation for 24 h at 37°C, the cells remaining on the upper surface of the membrane were removed with cotton swabs. The cells that migrated through the membrane were fixed with 4% paraformaldehyde and stained with a 0.1% crystal violet solution. Observations were made using an Olympus BX51 microscope (Olympus Corporation, Tokyo, Japan) with 10× magnification, and cell counting was performed manually. For the invasion assay, the procedure was similar, with the additional step of coating each well with Matrigel (Corning, Corning, NY, USA) for 24 h to allow solidification. To minimize observational bias, cell counting for migration and invasion assays was conducted manually by investigators blinded to the gravity conditions.

### Statistical Analysis

For gene expression analysis, DEGs between microgravity and normal gravity conditions were compared using an FC threshold of 1.5. Statistical significance was defined as a *p*-value < 0.05 or FDR < 0.2 using the Benjamini-Hochberg procedure. Hierarchical clustering was conducted using the Euclidean distance metric and complete linkage method. Functional annotation of DEGs was conducted using GO and KEGG via g:Profiler, with significantly enriched terms defined as adjusted *p*-values (padj) < 0.05, as provided by the g:Profiler tool.

GSEA was performed using software provided by the Broad Institute. The enrichment statistic was set to “weighted,” and the metric for ranking genes was “signal-to-noise.” Gene set size thresholds were set with a minimum of 15 genes and a maximum of 500 genes. The permutation type was “gene-set,” with the number of permutations set to 1000. Gene sets with an FDR < 0.25 were considered significantly enriched.

Statistical analysis was performed using a *t*-test to compare the number of migrated and invaded cells between the simulated microgravity and normal gravity conditions; statistical significance was set at a *p*-value < 0.05. Due to the small sample size (n = 3 per group), we did not conduct formal normality tests, as such tests have low power in small datasets. Although an independent *t*-test was applied to compare group means, the limited sample size may compromise the reliability of parametric assumptions. Therefore, the results should be interpreted with caution. The analysis was conducted using R version 4.3.1 (www.r-project.org).

## Results

### Differentially expressed genes analysis

**Figure 1:**
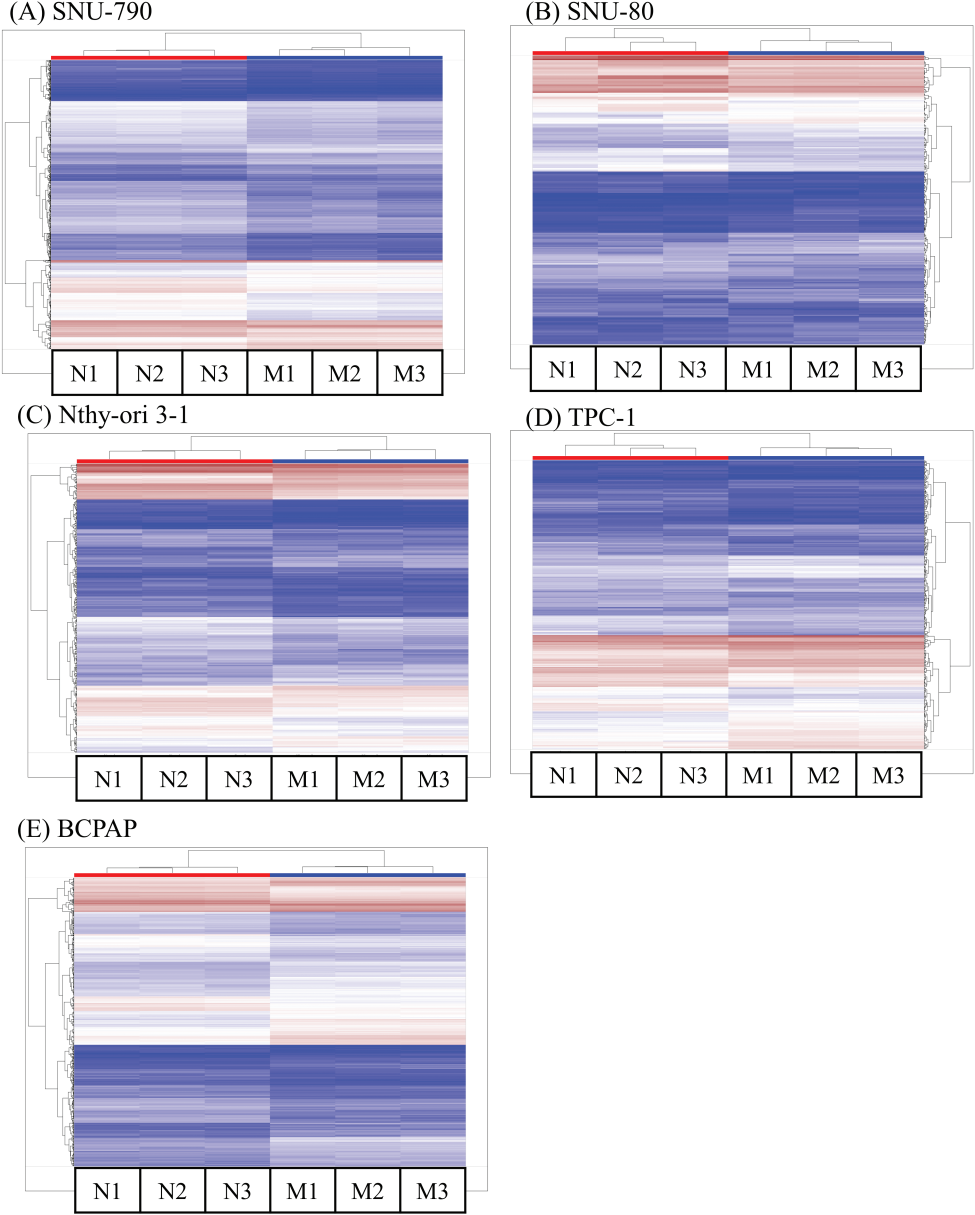
Hierarchical clustering analysis of differentially expressed genes (DEGs) in thyroid-related cell lines cultured under simulated microgravity. The first three columns (N1–N3) for each cell line represent normal gravity conditions, while the last three columns (M1–M3) depict microgravity conditions. (A) SNU-790. (B) SNU-80. (C) Nthy-ori 3-1. (D) TPC-1. (E) BCPAP. Upregulated DEGs are shown in brown, and downregulated DEGs are shown in blue.

**Figure 2:**
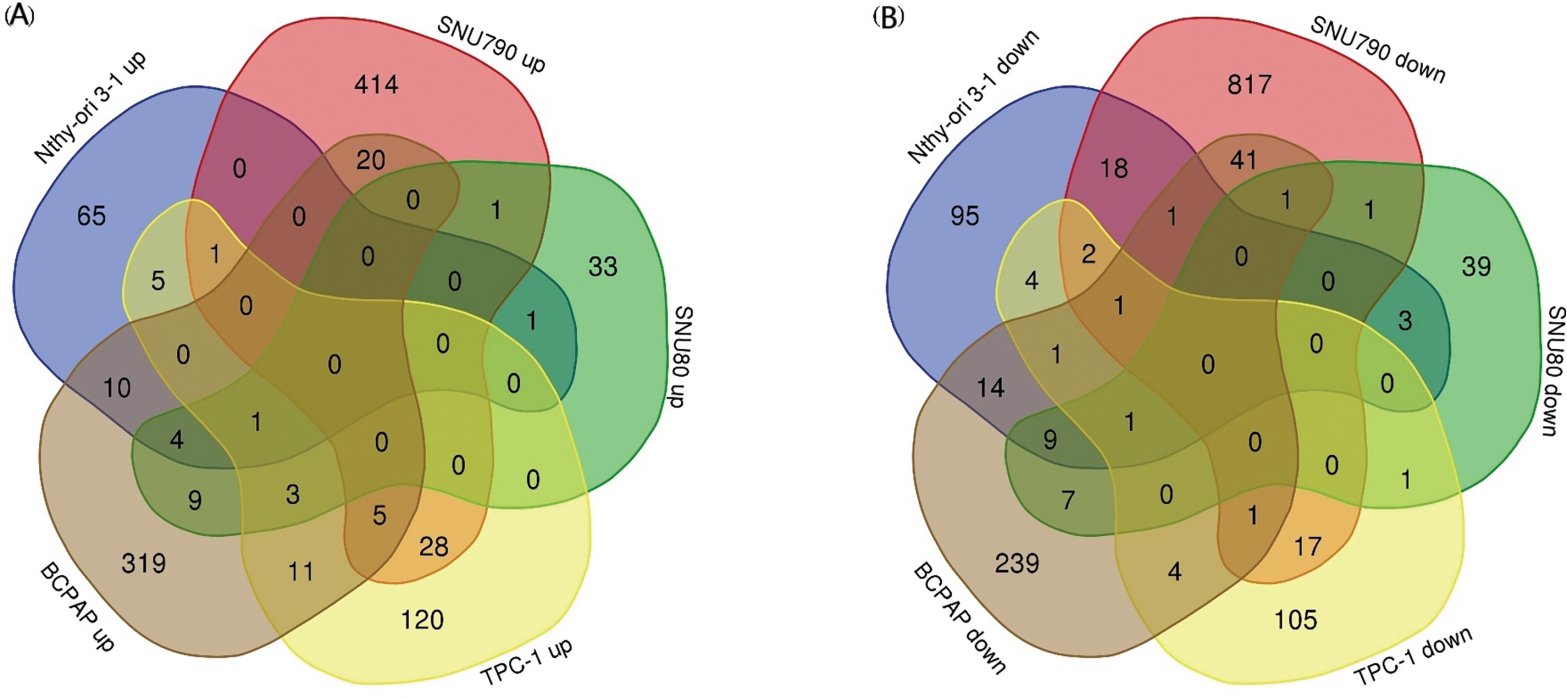
Common gene expression changes across five thyroid-related cell lines under simulated microgravity. (A) Venn diagram showing the common upregulation of differentially expressed genes (DEGs) across the five cell lines under microgravity. (B) Venn diagram showing the common downregulation of DEGs across the five cell lines under microgravity. The numbers within each section of the Venn diagrams represent the number of DEGs.

Tables A1–A5 summarize the top 20 upregulated and downregulated DEGs identified in each cell line cultured under microgravity conditions. DEG analysis showed that SNU-790 cells exhibited the most DEGs, with 2980 genes, followed by BCPAP (1033 genes), TPC-1 (562 genes), Nthy-ori 3-1 (477 genes), and SNU-80 (246 genes; Table 2). Hierarchical clustering analysis indicated that all cell lines exhibited distinct characteristics based on gravity conditions (Fig. 1). Fig. 2A,B, and Tables 3 and 4 present the common DEGs across the five cell lines. No DEGs were consistently upregulated or downregulated across all five cell lines. The PTC cell lines (SNU-790 and TPC-1) shared 34 upregulated and 18 downregulated DEGs. In particular, 13 genes related to histones were upregulated, while the cell cycle-related genes, such as *CDK1* (SNU-790, FC = 1.80, *p* = 0.001; TPC-1, FC = 1.70, *p* = 0.008), and *TTK* (SNU-790, FC = 1.50, *p* = 0.004; TPC-1, FC = 1.56, *p* = 0.003), were also upregulated. In the non-PTC cell lines, SNU-80 and BCPAP, 18 DEGs were commonly upregulated, and 16 were downregulated. The commonly upregulated genes in the non-PTC cell lines included *TLR3* (SNU-80, FC = 1.80, *p* < 0.001; BCPAP, FC = 1.61, *p* < 0.001; Nthy-ori 3-1, FC = 1.76, *p* < 0.001) and *GBP1* (SNU-80, FC = 1.62, *p* = 0.001; BCPAP, FC = 2.25, *p* < 0.001; Nthy-ori 3-1, FC = 2.43, *p* < 0.001). The downregulated genes included *CA9*, associated with hypoxia (SNU-80, FC = −3.70, *p* = 0.035; BCPAP, FC = −7.22, *p* < 0.001; Nthy-ori 3-1, FC = −2.47, *p* < 0.001), along with genes involved in glucose metabolism, such as *ENO2*, *HK2*, and *PFKFB4*, and *PDK1* (SNU-80, FC = −2.52, *p* = 0.038; BCPAP, FC = −3.10, *p* < 0.001).

**Table 2:** Number of differentially expressed genes in five thyroid-related cell lines under simulated microgravity compared to normal gravity conditions

Cell lines	DEGs (n)	Up-regulated DEGs (n (%))	Down-regulated DEGs (n (%))
**SNU-790**	2980	777 (26.07%)	2203 (73.93%)
**SNU-80**	246	130 (52.85%)	116 (47.15%)
**Nthy-ori 3-1**	477	164 (34.38%)	313 (65.62%)
**TPC-1**	562	245 (43.59%)	317 (56.41%)
**BCPAP**	1033	520 (50.34%)	513 (49.66%)

**Table 3:** Commonly differentially expressed genes in the papillary thyroid cancer cell lines (SNU-790 and TPC-1) cultured under simulated microgravity compared to normal gravity. Genes with *p* < 0.05 are shown, sorted by absolute fold change in SNU-790

DEGs	SNU-790	TPC-1	Description
*HIST1H3B*	3.58	1.75	Histone cluster 1, H3b
*HIST1H3G*	3.49	2.13	Histone cluster 1, H3g
*HIST2H2AB*	2.57	1.91	Histone cluster 2, H2ab
*HIST1H1B*	2.42	1.8	Histone cluster 1, H1b
*CXCL8*	2.35	2.04	Chemokine (C-X-C motif) ligand 8
*HIST1H2AI*	2.17	2.07	Histone cluster 1, H2ai
*RNVU1-6*	2.03	1.55	RNA, variant U1 small nuclear 6
*RNU5D-1*	1.94	1.57	RNA, U5D small nuclear 1
*HIST2H3A*	1.87	1.65	Histone cluster 2, H3a
*HIST2H3C*	1.87	1.65	Histone cluster 2, H3c
*CDK1*	1.80	1.70	Cyclin-dependent kinase 1
*SPC24*	1.77	1.58	SPC24, NDC80 kinetochore complex component
*CKAP2L*	1.77	1.5	Cytoskeleton-associated protein 2-like
*UBE2S*	1.76	1.71	Ubiquitin-conjugating enzyme E2S
*CENPN*	1.75	1.74	Centromere protein N
*SNORA71D*	1.75	1.62	Small nucleolar RNA, H/ACA box 71D
*HIST1H2AE*	1.71	1.81	Histone cluster 1, H2ae
*HIST1H2BB*	1.69	1.95	Histone cluster 1, H2bb
*HIST1H4D*	1.68	1.71	Histone cluster 1, H4d
*RNU5B-1*	1.68	1.56	RNA, U5B small nuclear 1
*ATF3*	1.66	2.91	Activating transcription factor 3
*HIST1H2BI*	1.64	1.75	Histone cluster 1, H2bi
*PBK*	1.64	1.66	PDZ binding kinase
*HIST1H4A*	1.64	1.53	Histone cluster 1, H4a
*ESCO2*	1.60	1.61	Establishment of sister chromatid cohesion N-acetyltransferase 2
*HIST1H3F*	1.56	1.65	Histone cluster 1, H3f
*TK1*	1.56	1.61	Thymidine kinase 1, soluble
*SKA3*	1.55	1.66	Spindle and kinetochore-associated complex subunit 3
*IL1B*	1.55	1.51	Interleukin 1 beta
*PRR11*	1.54	1.62	Proline rich 11
*DLGAP5*	1.54	1.61	Discs, large (Drosophila) homolog-associated protein 5
*SHANK1*	1.53	1.62	SH3 and multiple ankyrin repeat domains 1
*NUSAP1*	1.52	1.54	Nucleolar and spindle-associated protein 1
*TTK*	1.50	1.56	TTK protein kinase
*MIRLET7C*	−2.06	−1.68	MicroRNA let-7c
*ZNF280D*	−1.90	−1.76	Zinc finger protein 280D
*CFI*	−1.85	−1.60	Complement factor I
*C9orf3*	−1.84	−2.06	Chromosome 9 open reading frame 3
*FER1L4*	−1.74	−1.56	Fer-1-like family member 4, pseudogene (functional)
*TXNIP*	−1.68	−1.59	Thioredoxin interacting protein
*ADAM20P1*	−1.68	−1.52	ADAM metallopeptidase domain 20 pseudogene 1
*PLEKHH2*	−1.63	−1.86	Pleckstrin homology domain containing, family H (with MyTH4 domain) member 2
*MORC3*	−1.60	−1.82	MORC family CW-type zinc finger 3
*CHAF1B*	−1.60	−1.82	Chromatin assembly factor 1, subunit B (p60)
*TGFB2-OT1*	−1.57	−1.59	TGFB2 overlapping transcript 1
*TGFB2*	−1.57	−1.59	Transforming growth factor beta 2
*C1orf132*	−1.54	−1.76	Chromosome 1 open reading frame 132
*MIR580*	−1.53	−1.69	MicroRNA 580
*ADAMTS9-AS2*	−1.52	−1.68	ADAMTS9 antisense RNA 2
*MIR23B*	−1.51	−2.06	MicroRNA 23b
*PCDHB7*	−1.51	−1.65	Protocadherin beta 7
*MIR27B*	−1.51	−1.58	MicroRNA 27b

Notes: DEGs, differentially expressed genes; The genes highlighted in red indicate those identified as significant in functional annotation analysis.

**Table 4:** Common differentially expressed genes in the non-papillary thyroid cancer cell lines (Nthy-ori 3-1, SNU-80, and BCPAP) cultured under simulated microgravity compared to normal gravity. Genes with *p* < 0.05 are shown, sorted by absolute fold change in SNU-80

DEGs	SNU-80	BCPAP	Nthy-ori 3-1	Description
*GBP4*	2.02	6.24	n/a	Guanylate binding protein 4
*MIR4668*	1.97	2.92	n/a	MicroRNA 4668
*IFI44L*	1.94	1.94	1.66	Interferon-induced protein 44-like
*TLR3*	1.80	1.61	1.76	Toll-like receptor 3
*HERC6*	1.68	1.81	n/a	HECT and RLD domain containing E3 ubiquitin protein ligase family member 6
*IFIT1*	1.66	3.20	n/a	Interferon-induced protein with tetratricopeptide repeats 1
*NQO1*	1.65	1.57	1.6	NAD(P)H dehydrogenase, quinone 1
*IFIH1*	1.62	2.26	n/a	Interferon-induced, with helicase C domain 1
*GBP1*	1.62	2.25	2.43	Guanylate binding protein 1, interferon-inducible
*TAF9B*	1.62	1.89	1.58	TAF9B RNA polymerase II, TATA box binding protein (TBP)-associated factor, 31kDa
*PLA2G4A*	1.59	1.66	n/a	Phospholipase A2, group IVA (cytosolic, calcium-dependent)
*XAF1*	1.56	1.87	n/a	XIAP associated factor 1
*RAB27B*	1.56	1.84	n/a	RAB27B, member RAS oncogene family
*SERPINB4*	1.52	1.76	n/a	Serpin peptidase inhibitor, clade B (ovalbumin), member 4
*SERPINB3*	1.52	1.76	n/a	Serpin peptidase inhibitor, clade B (ovalbumin), member 3
*JAKMIP2*	1.51	1.65	n/a	Janus kinase and microtubule interacting protein 2
*CA9*	−3.70	−7.22	−2.47	Carbonic anhydrase IX
*PDK1*	−2.52	−3.10	n/a	Pyruvate dehydrogenase kinase, isozyme 1
*TMEM45A*	−2.26	−2.33	−1.71	Transmembrane protein 45A
*VLDLR*	−2.21	−1.76	n/a	Very low-density lipoprotein receptor
*NDRG1*	−2.15	−2.25	−2.43	N-myc downstream regulated 1
*LOX*	−2.12	−4.15	−2.02	Lysyl oxidase
*COL11A1*	−2.08	−1.64	n/a	Collagen, type XI, alpha 1
*ENO2*	−2.03	−2.53	−1.59	Enolase 2 (gamma, neuronal)
*HK2*	−1.81	−2.39	−1.68	Hexokinase 2
*GBE1*	−1.81	−1.97	−1.66	Glucan (1,4-alpha-), branching enzyme 1
*AK4*	−1.79	−2.09	n/a	Adenylate kinase 4
*PPFIA4*	−1.67	−2.18	−1.82	Protein tyrosine phosphatase, receptor type, f polypeptide (PTPRF), interacting protein (liprin), alpha 4
*INSIG2*	−1.66	−1.97	−1.53	Insulin-induced gene 2
*PFKFB4*	−1.64	−1.67	−1.58	6-phosphofructo-2-kinase/fructose-2,6-biphosphatase 4
*MIR329-1*	−1.62	−1.70	n/a	MicroRNA 329-1
*CTGF*	−1.57	−1.98	n/a	Connective tissue growth factor
*CCL28*	−1.55	−1.79	n/a	Chemokine (C-C motif) ligand 28
*ARFGEF3*	−1.51	−1.53	n/a	ARFGEF family member 3

Notes: DEGs, differentially expressed genes; The genes highlighted in red were identified as significant in functional annotation analysis. Differentially expressed genes in Nthy-ori 3-1 with no significant expression differences are marked as n/a.

### Functional Annotation Analysis

**Figure 3:**
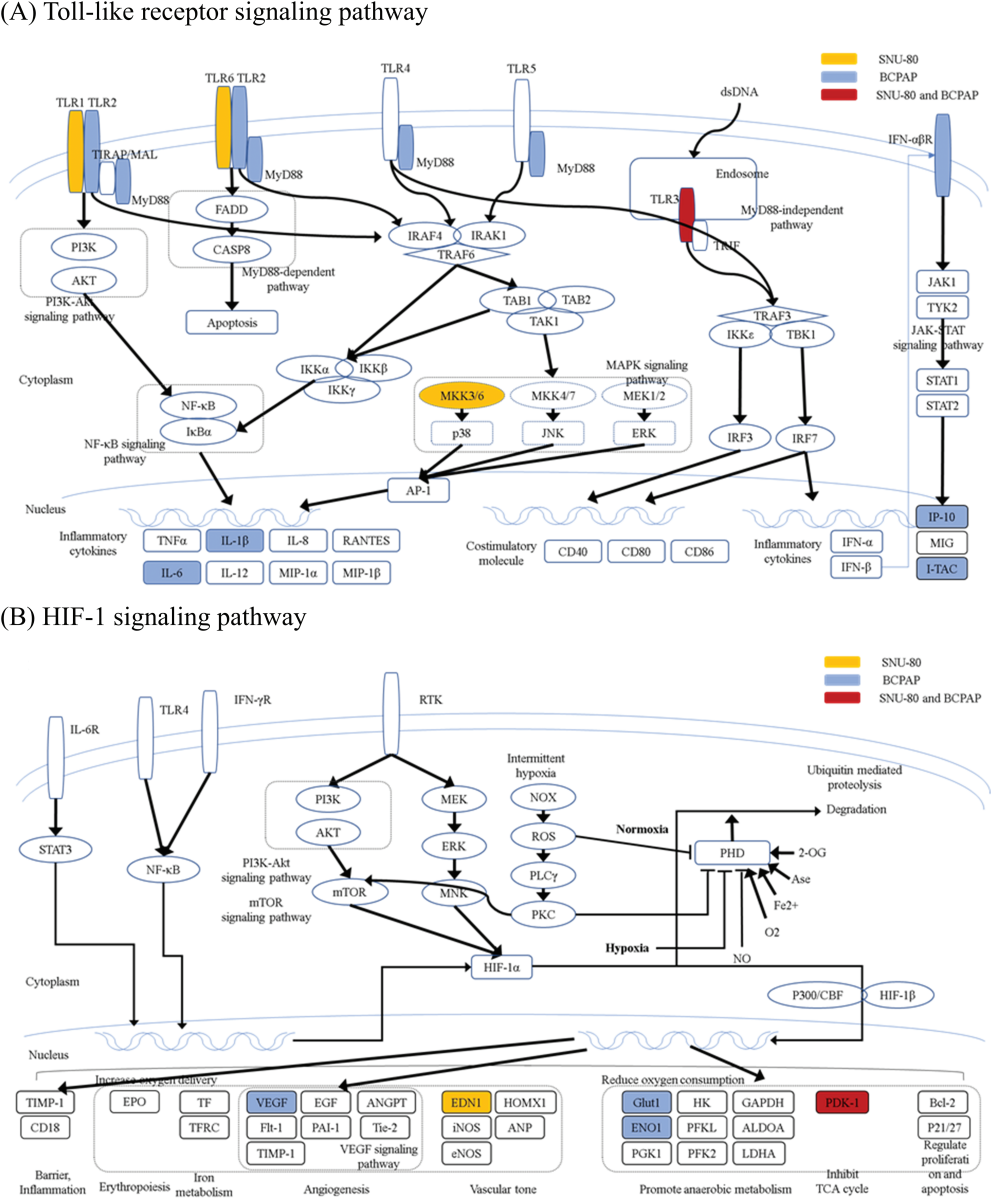
KEGG pathway enrichment analysis of differentially expressed genes in SNU-80 and BCPAP cells cultured under simulated microgravity compared to normal gravity. (A) KEGG enrichment analysis for upregulated DEGs in SNU-80 and BCPAP cell lines under microgravity. (B) KEGG enrichment analysis for downregulated DEGs in SNU-80 and BCPAP cell lines under microgravity.

**Figure 4:**
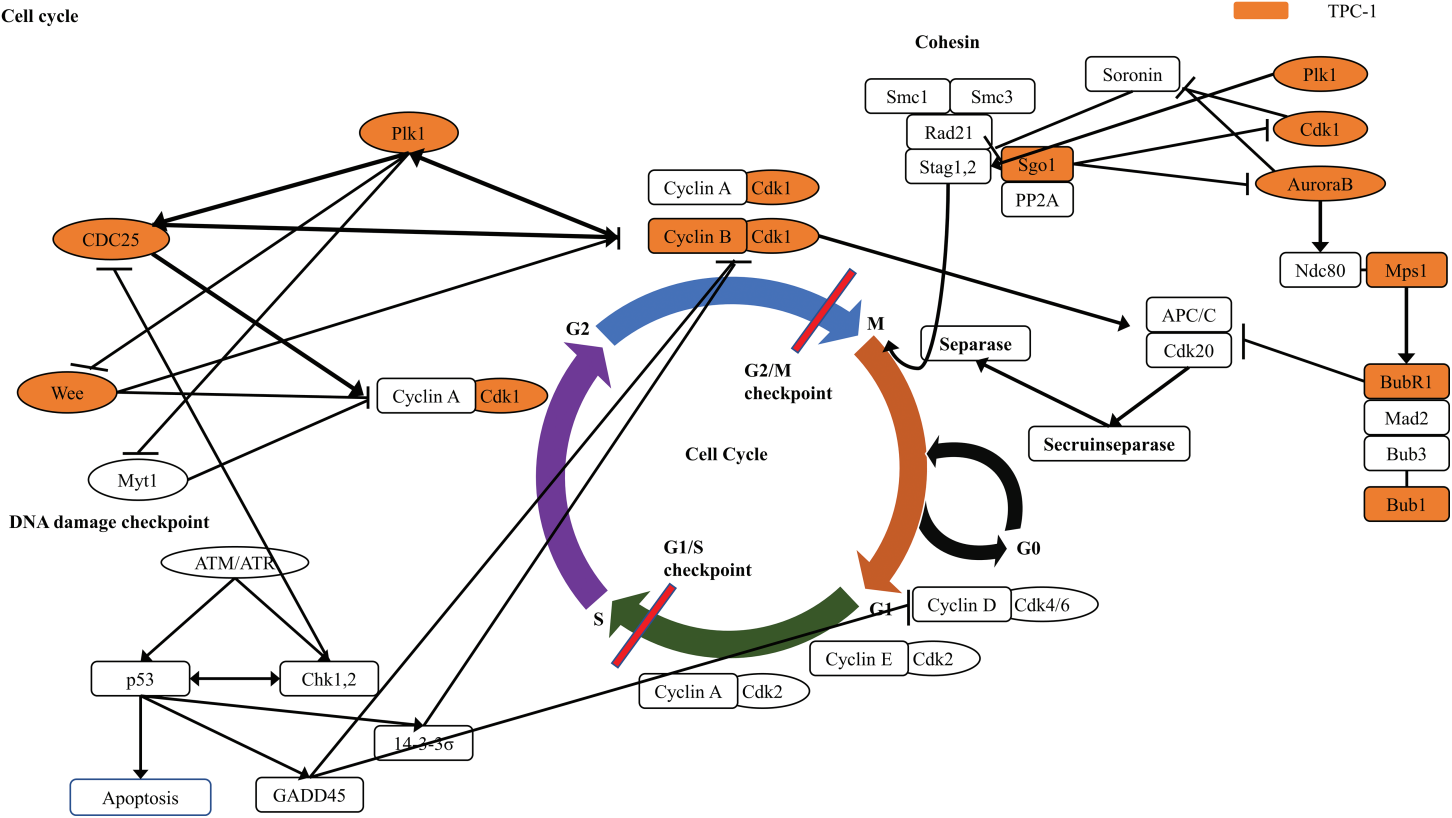
KEGG pathway enrichment analysis of upregulated differentially expressed genes in TPC-1 cells cultured under simulated microgravity compared to normal gravity.

**Figure A1:**
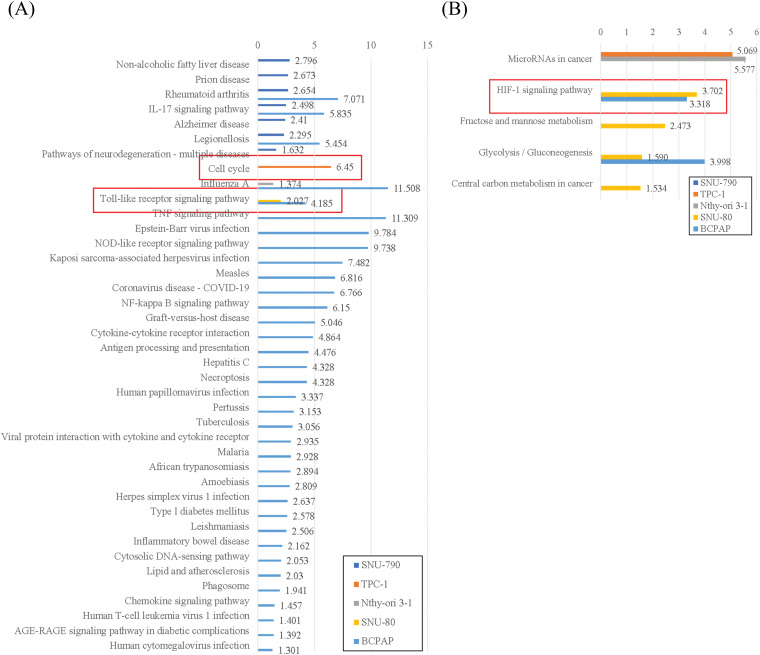
KEGG enrichment analysis of differentially expressed genes in five thyroid-related cell lines cultured under microgravity compared to normal gravity. (A) KEGG enrichment analysis of upregulated differentially expressed genes. (B) KEGG enrichment analysis of down-regulated differentially expressed genes. All results are negative values of log_10_ of adjusted *p*-value. The red box shows the significant findings of the study

Figs. 3, 4 and A1 present the results of the KEGG pathway analysis. The DEGs commonly upregulated in SNU-80 and BCPAP cells cultured under simulated microgravity were associated with the Toll-like receptor (TLR) signaling pathway (KEGG:04620; SNU-80 -log_10_ (adjusted *p*-value (*p*_*adj*_)) = 2.027, BCPAP -log_10_(*p*_*adj*_) = 4.185, Fig. 3A). In addition, the downregulated DEGs in these cells were linked to the hypoxia-inducible factor (HIF)-1 signaling pathway (KEGG:04066; SNU-80 -log_10_(*p*_*adj*_) = 3.702, BCPAP -log_10_(*p*_*adj*_) = 3.318, Fig. 3B) and the glycolysis/gluconeogenesis pathway (KEGG:00010; SNU-80 -log_10_(*p*_*adj*_) = 1.590, BCPAP -log_10_(*p*_*adj*_) = 3.998). In the case of SNU-790 and TPC-1, no KEGG terms were commonly enriched. In TPC-1, however, the upregulated genes under microgravity were associated with the cell cycle pathway (KEGG:04110; -log_10_(*p*_*adj*_) = 6.450). These upregulated DEGs were related to the S, G2, and M phases (Fig. 4).

**Figure 5:**
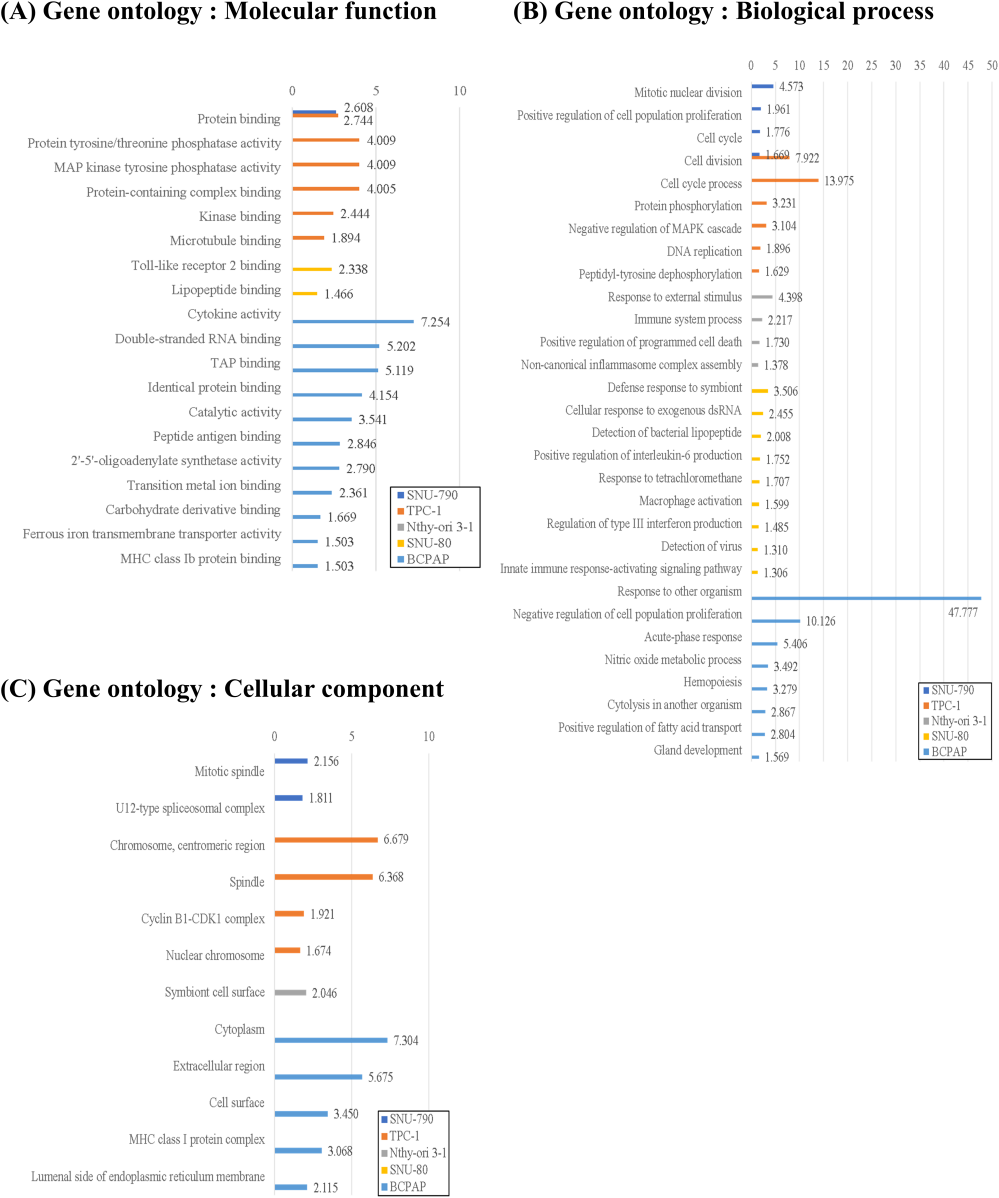
Gene Ontology (GO) enrichment analysis of upregulated differentially expressed genes (DEGs) in thyroid-related cell lines cultured under simulated microgravity compared to normal gravity. (A) GO enrichment analysis for upregulated DEGs in terms of the molecular function. (B) GO enrichment analysis for upregulated DEGs in terms of the biological process. (C) GO enrichment analysis for upregulated DEGs in terms of the cellular components. All results are presented as the negative log_10_ of the adjusted *p*-value.

Fig. 5 shows the GO enrichment analysis of upregulated DEGs in SNU-790 and TPC-1 cells cultured under simulated microgravity. In terms of molecular function, both cell lines showed enrichment in protein binding (GO:0005515, SNU-790 -log_10_(*p*_***adj***_) = 2.608 and TPC-1 -log_10_(*p*_***adj***_) = 2.744, Fig. 5A). Regarding biological processes, the upregulated genes were associated with mitotic nuclear division (GO:0140014, SNU-790, -log_10_(*p*_***adj***_) = 4.573) and cell division (GO:0051301, TPC-1, -log_10_(*p*_***adj***_) 7.922, Fig. 5B). In the cellular component category, these DEGs were related to the mitotic spindle (GO:0072686, SNU-790 -log_10_(*p*_***adj***_) = 2.156) and spindle (GO:0005819, TPC-1 -log_10_(*p*_***adj***_) = 6.368, Fig. 5C). For the non-PTC cell lines, the upregulated DEGs were linked primarily to molecular function related to Toll-like receptor 2 binding (GO:0035663, SNU-80, -log_10_(*p*_***adj***_) = 2.338) and the cytokine activity (GO:0005125, BCPAP, -log_10_(*p*_***adj***_) = 7.254; Fig. 5A). In terms of biological processes, these DEGs were enriched in pathways associated with the cellular response to external stimuli, including the cellular response to external stimuli (response to external stimulus, GO:0009605, Nthy-ori 3-1 -log_10_(*p*_***adj***_) = 4.398; the cellular response to exogenous dsRNA, GO:0071360, SNU-80, -log_10_(*p*_***adj***_) = 2.455, and the response to other organisms, GO:0051707, BCPAP -log_10_(*p*_***adj***_) = 47.777; Fig. 5B). In the cellular component category, no significant enrichment was observed in SNU-80, whereas in BCPAP, upregulated DEGs were significantly associated with extracellular region (GO:0005576, -log_10_(*p*_***adj***_) = 5.675), cell surface (GO:0009986, -log_10_(*p*_***adj***_) = 3.450), and MHC class I protein complex (GO:0042612, -log_10_(*p*_***adj***_) = 3.068; Fig. 5C).

**Figure 6:**
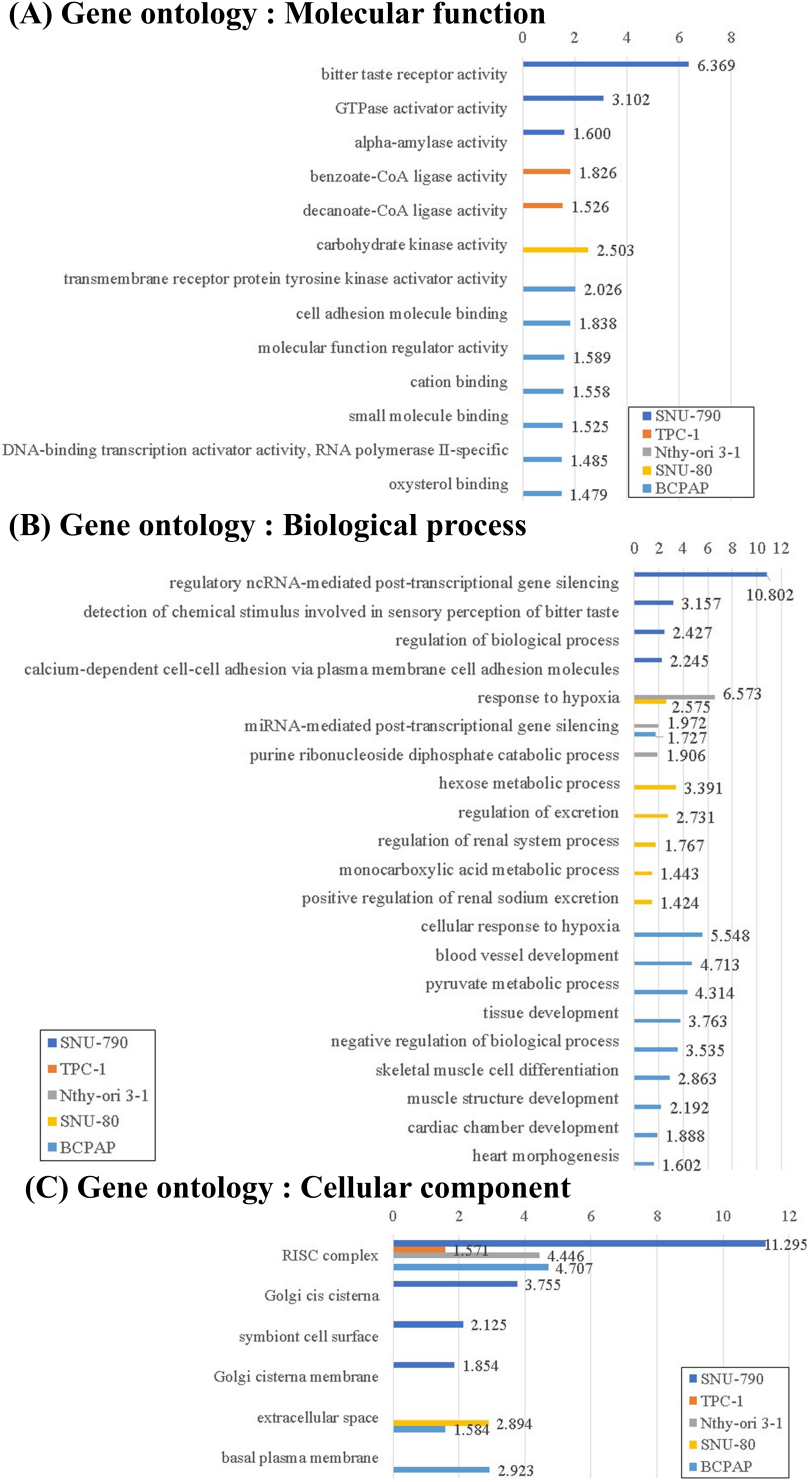
Gene Ontology (GO) enrichment analysis of downregulated differentially expressed genes (DEGs) in thyroid-related cell lines cultured under simulated microgravity compared to normal gravity. (A) GO enrichment analysis for downregulated DEGs in terms of the molecular function. (B) GO enrichment analysis for downregulated DEGs in terms of the biological process. (C) GO enrichment analysis for downregulated DEGs in terms of the cellular components. All results are presented as the negative log_10_ of the adjusted *p*-value.

The downregulated DEGs under microgravity were associated with molecular functions such as DNA-binding transcription activator activity, RNA polymerase II–specific (GO:0001228, BCPAP, log_10_(*p*_***adj***_) = 1.485; Fig. 6A). The downregulated DEGs in non-PTC cells under microgravity were related to the responses to hypoxia (response to hypoxia, GO:0001666, Nthy-ori 3-1, -log_10_(*p*_***adj***_) = 6.573; SNU-80, -log_10_(*p*_***adj***_) = 2.575; and the cellular response to hypoxia, GO:0071456, BCPAP, -log_10_(*p*_***adj***_) = 5.548; Fig. 6B). Furthermore, downregulated DEGs in both BCPAP and SNU-80 were significantly associated with the extracellular space (GO:0005615, SNU-80, -log_10_(*p*_***adj***_) = 1.384; and BCPAP, -log_10_(*p*_***adj***_) = 2.894; Fig. 6C).

**Figure 7:**
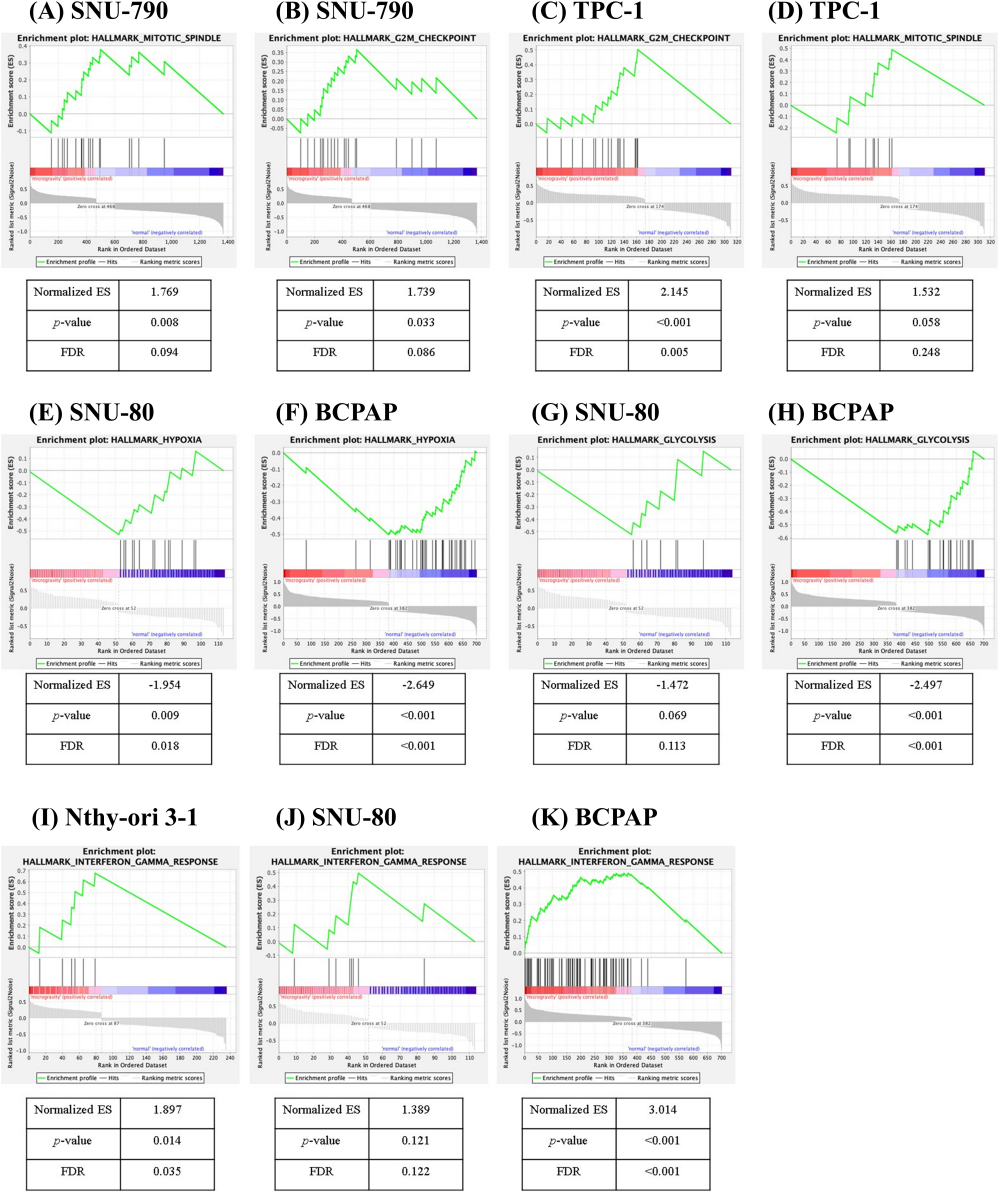
Gene set enrichment analysis of differentially expressed genes in thyroid-related cell lines cultured under simulated microgravity compared to normal gravity. (A, B) SNU-790, (C, D) TPC-1, (E) SNU-80, (F) BCPAP, (G) SNU-80, (H) BCPAP, (I) Nthy-ori 3-1, (J) SNU-80, and (K) BCPAP. ES denotes the enrichment score, and FDR refers to the false discovery rate.

Fig. 7 presents the GSEA results for the five different cell types. No gene sets were commonly enriched across all five cell lines. In SNU-790 cells, gene sets related to the mitotic spindle (normalized enrichment score (NES) = 1.769, *p* = 0.008, FDR = 0.094, Fig. 7A) and G2-M checkpoint (NES = 1.739, *p* = 0.033, FDR = 0.086, Fig. 7B) showed a positive correlation with the microgravity environment. For TPC-1, the G2-M checkpoint (NES = 2.145, *p* < 0.001, FDR = 0.005, Fig. 7C) and mitotic spindle (NES = 1.532, *p* = 0.058, FDR = 0.248, Fig. 7D). Gene sets were also positively correlated with microgravity. In SNU-80 and BCPAP cells, the gene sets related to hypoxia (NES = −1.954, *p* = 0.009, FDR = 0.018, and NES = −2.497, *p* < 0.001, FDR < 0.001, respectively; Figs. 7E, 7F) and glycolysis (NES = −1.472, *p* = 0.069, FDR = 0.113, and NES = −2.649, *p* < 0.001, FDR < 0.001, respectively; Fig. 7G,H) were negatively correlated with microgravity. In addition, the interferon (IFN)-γ response gene sets showed a positive correlation with microgravity in Nthy-ori 3-1 (NES = 1.897, *p* = 0.014, FDR = 0.035, Fig. 7I), SNU-80 (NES = 1.389, *p* = 0.121, FDR = 0.122, Fig. 7J), and BCPAP cells (NES = 3.014, *p* < 0.001, FDR < 0.001, Fig. 7K).

### Transwell migration and invasion assay

**Figure 8:**
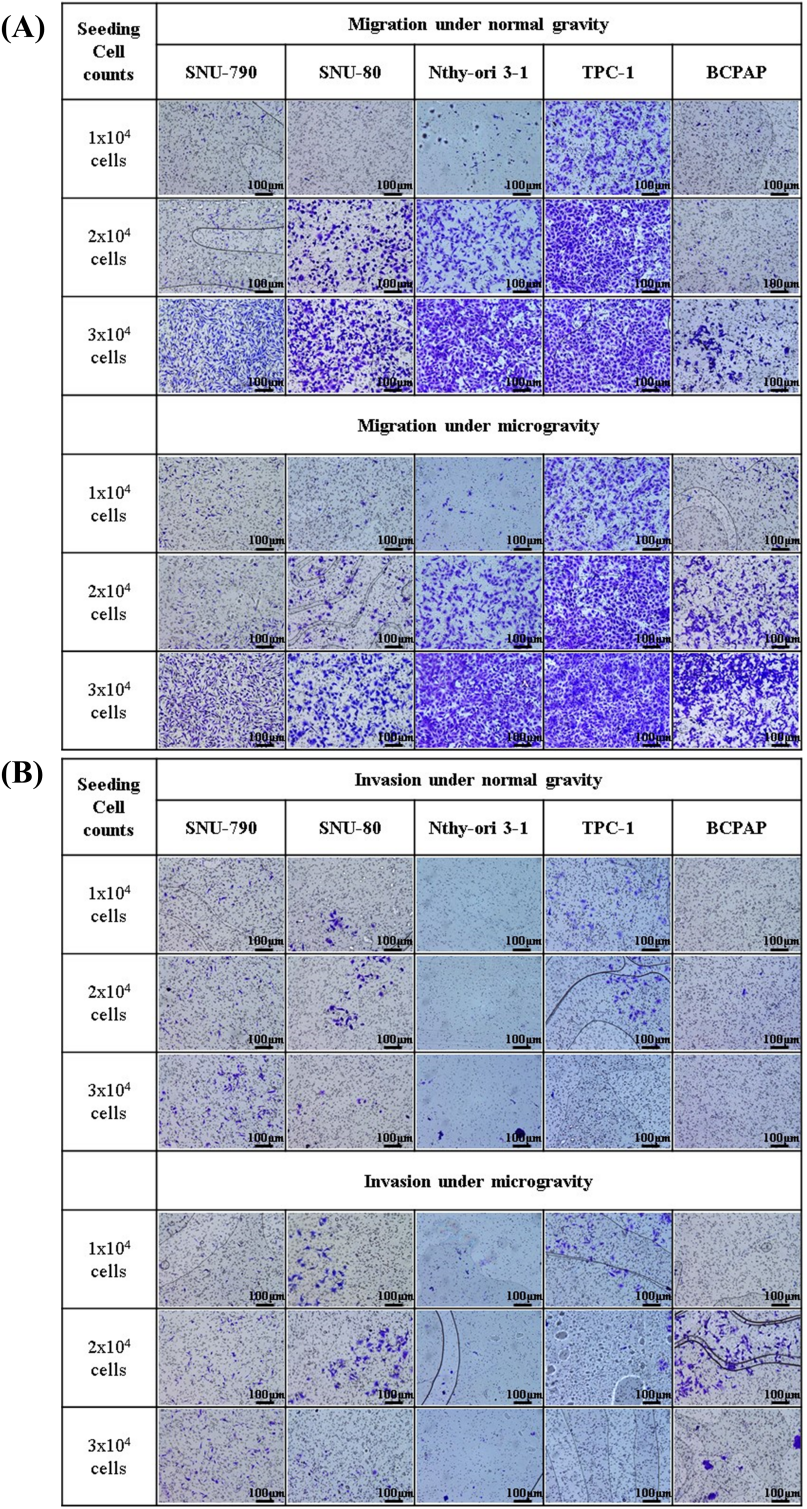
Microscopic images of transwell migration and invasion assays of thyroid-related cell lines under simulated microgravity and normal gravity conditions. After a five-day cultivation period, five different cell lines were seeded at densities of 1 × 10^4^, 2 × 10^4^, and 3 × 10^4^ cells per well. (A) Representative images of the transwell migration assay for each of the five cell lines (SNU-790, SNU-80, Nthy-ori 3-1, TPC-1, and BCPAP) under microgravity and normal gravity conditions at varying seeding densities. (B) Representative images of the transwell invasion assay under the same conditions as in (A), using Matrigel-coated inserts to assess invasive capacity. All images were captured at ×10 magnification. Scale bars represent 100 μm.

**Figure 9:**
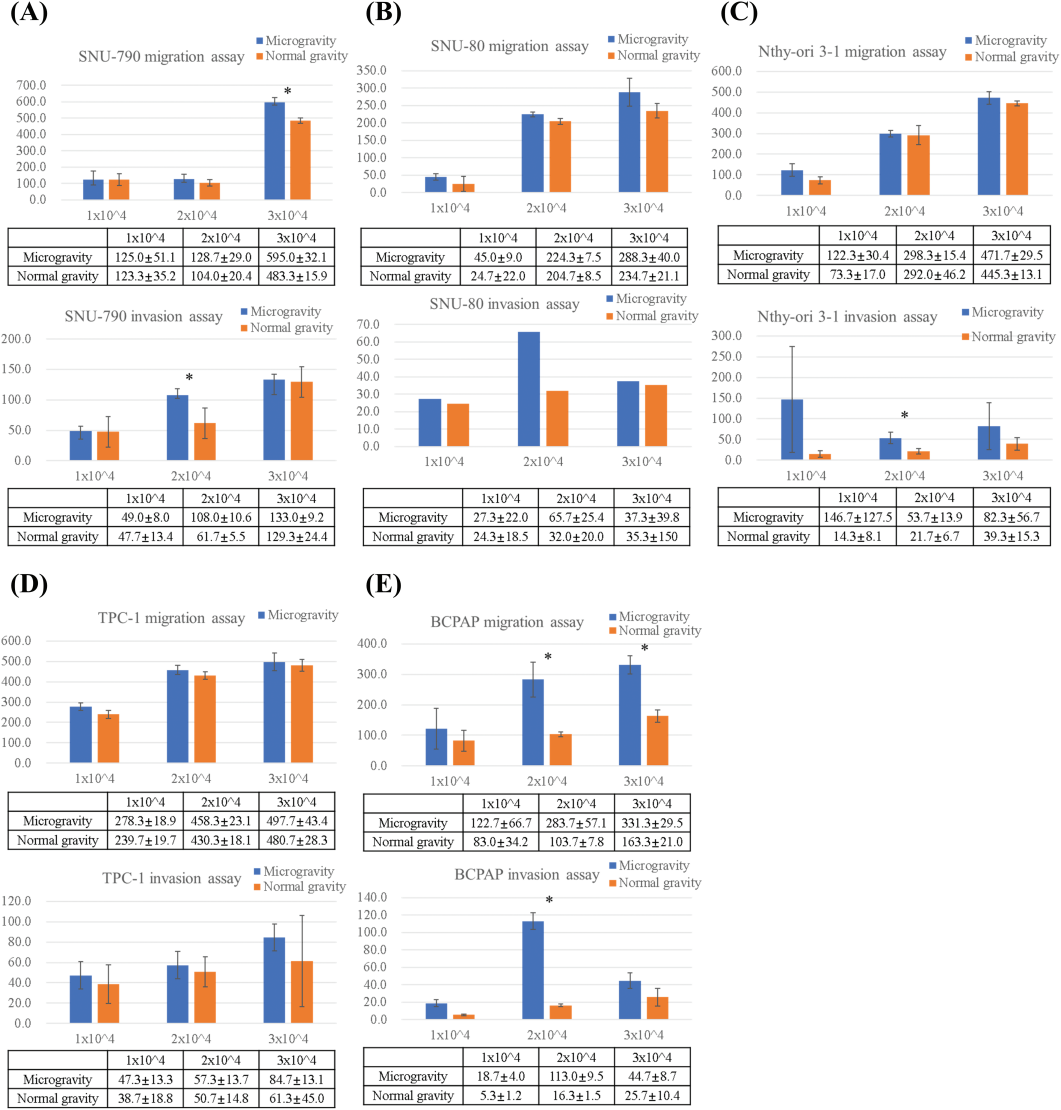
Comparison of migrated and invaded cell counts by transwell migration and invasion assays of thyroid-related cell lines under simulated microgravity and normal gravity conditions. After a five-day cultivation period, five different cell lines were seeded at densities of 1 × 10^4^, 2 × 10^4^, and 3 × 10^4^ cells per well. Quantitative comparative analysis of migration and invasion assays across five different thyroid-related cell lines: (A) SNU-790, (B) SNU-80, (C) Nthy-ori 3-1, (D) TPC-1, and (E) BCPAP. *Y*-axis indicates the number of cells counted. * indicate statistically significant differences with a *p* < 0.05.

Fig. 8 and 9 present the results of the transwell migration and invasion assays. In the SNU-790 cell line, migration was significantly increased under microgravity at a seeding density of 3 × 10^4^ cells (microgravity: 660.0 ± 25.1 vs. normal gravity: 509.0 ± 32.1; *p* < 0.05). Invasion was also significantly higher under microgravity at 2 × 10^4^ cells (108.0 ± 9.6) compared to normal gravity (67.5 ± 15.5; *p* < 0.05). At 1 × 10^4^ and 2 × 10^4^ cells in the migration assay (125.0 ± 15.2 vs. 123.5 ± 13.2; 128.7 ± 16.0 vs. 100.6 ± 14.4) and at 1 × 10^4^ and 3 × 10^4^ cells in the invasion assay (49.0 ± 8.4 vs. 47.7 ± 11.8; 139.0 ± 18.3 vs. 125.9 ± 24.4), no statistically significant differences were observed (Fig. 9A).

In SNU-80 cells, migration under microgravity was 45.0 ± 9.2, 220.3 ± 19.5, and 288.1 ± 28.7 at 1 × 10^4^, 2 × 10^4^, and 3 × 10^4^ cells, respectively, compared to 28.7 ± 8.4, 204.7 ± 28.5, and 224.4 ± 25.3 under normal gravity. Invasion values were 27.3 ± 5.1, 65.7 ± 8.1, and 36.5 ± 7.2 under microgravity versus 23.6 ± 4.6, 30.5 ± 9.8, and 33.2 ± 6.1 under normal gravity, respectively. None of these differences were statistically significant (Fig. 9B).

In Nthy-ori 3-1 cells, invasion at 2 × 10^4^ cells was significantly increased under microgravity (83.1 ± 9.2) compared to normal gravity (32.8 ± 5.7) (*p* < 0.05). Migration values at 1 × 10^4^, 2 × 10^4^, and 3 × 10^4^ cells were 122.3 ± 9.5, 286.1 ± 14.7, and 471.7 ± 29.5 under microgravity, versus 86.7 ± 8.4, 268.1 ± 17.2, and 460.3 ± 27.4 under normal gravity. Invasion at 1 × 10^4^ and 3 × 10^4^ was 145.7 ± 98.5 and 73.6 ± 12.3 under microgravity, compared to 18.7 ± 5.2 and 30.9 ± 15.3 under normal gravity. These differences were not statistically significant (Fig. 9C).

For TPC-1 cells, migration under microgravity was 279.7 ± 18.3, 459.8 ± 20.4, and 509.2 ± 21.5 at 1 × 10^4^, 2 × 10^4^, and 3 × 10^4^ cells, respectively, compared to 239.9 ± 17.2, 448.3 ± 22.4, and 487.6 ± 20.8 under normal gravity. Invasion values were 43.7 ± 10.1, 51.3 ± 12.2, and 84.3 ± 13.5 under microgravity versus 33.5 ± 12.8, 47.8 ± 10.4, and 61.7 ± 18.6 under normal gravity. No statistically significant differences were observed (Fig. 9D).

In the BCPAP cell line, a significant increase in migration was observed under microgravity at both 2 × 10^4^ and 3 × 10^4^ cells (2 × 10^4^: 283.5 ± 7.1 vs. 179.0 ± 9.5; 3 × 10^4^: 331.3 ± 29.5 vs. 174.3 ± 8.6; *p* < 0.05). At 1 × 10^4^ cells, migration was 122.7 ± 16.4 under microgravity and 80.3 ± 14.2 under normal gravity; this difference was not statistically significant. Invasion was also significantly higher under microgravity at 2 × 10^4^ cells (113.9 ± 5.1 vs. 63.0 ± 4.3) (*p* < 0.05). At 1 × 10^4^ and 3 × 10^4^ cells, invasion values were 18.7 ± 4.2 and 45.7 ± 8.7 under microgravity, and 13.6 ± 3.1 and 25.7 ± 10.4 under normal gravity, respectively, with no statistically significant differences (Fig. 9E).

## Discussion

As space exploration advances, it is essential to understand how microgravity affects radiosensitive organs like the thyroid. While previous studies have explored thyroid cancer cell behavior in microgravity, most focused on a single cell line and lacked functional analysis. To address this, we investigated five thyroid-related cell lines—including those from Korean populations and PTC—and assessed both gene expression and cell aggressiveness using transcriptomic profiling and transwell migration and invasion assays under simulated microgravity. Our findings revealed distinct gene expression patterns when five different thyroid-related cell lines were cultured in microgravity. In the present study, the genes associated with the cell cycle (SNU-790 and TPC-1) were upregulated, and the genes related to the HIF-1 signaling pathway (SNU-80 and BCPAP) were downregulated. The upregulated genes were linked to the innate immune system (SNU-80, BCPAP, and Nthy-ori 3-1). To the best of the authors’ knowledge, this is the first study to investigate the properties of five different thyroid cell lines in a microgravity environment, providing a comprehensive perspective on how simulated microgravity impacts thyroid cells.

This study found no common DEG changes among the five thyroid cell lines. Notably, the ATC and PDTC cells exhibited DEG expression patterns similar to those of normal thyrocytes, while the PTC cells displayed distinct characteristics. The ATC and PDTC cells have poorly differentiated features, lose normal thyroid characteristics, and are associated with invasiveness and poor prognosis [2]. Mutations in genes, such as *TP53* and *TERT* promoter, are more prevalent in ATC and PDTC, contributing to their higher mutation burden compared to well-differentiated thyroid cancer. These mutations activate various downstream signaling pathways, significantly increasing the hazard ratio for overall survival (*TERT* promoter mutation 1.492, and *TP53* alteration 2.205) [22,23]. Previous studies reported that various cancer cells and stem cells undergo differentiation and redifferentiation under microgravity conditions [24]. Therefore, microgravity may have a more pronounced effect on ATC and PDTC cells, which have a higher genetic alteration burden. This could explain why ATC and PDTC cells showed DEG expression patterns more closely to those of normal thyrocytes.

Mechanotransduction is the process where changes in mechanical forces, such as gravity, are perceived as danger signals and are converted into biosignals that trigger immune responses [25]. In the present study, the overexpression of genes related to TLRs was observed when SNU-80, BCPAP, and Nthy-ori 3-1 cells were cultured in a microgravity environment. TLRs function as pathogen recognition receptors, identifying pathogen-associated molecular patterns and activating the innate immune system [26]. This activation promotes cytokine release and modulates the adaptive immune system [27]. However, it is well known that microgravity adversely affects the human immune system. Various studies have shown that microgravity can cause abnormalities in adaptive immunity, leading to latent virus reactivation and allergic reactions [28,29]. One study analyzing human peripheral blood mononuclear cells in microgravity reported that cytoskeletal changes impair the functional immune response, even though changes in gene expression related to acute immune response and chemokines were observed [29]. Interestingly, the immune activation induced by microgravity varies over time. In CTLA-4 cells, short-term exposure (24 h) increased IL-2 activity, whereas prolonged culture (96–120 h) led to reduced IL-2 production and T-cell exhaustion, suggesting a shift from immune activation to suppression over time [30]. T-cell exhaustion is one of the immunological states associated with aging, indicating that prolonged exposure to microgravity may eventually lead to an immunosuppressive state [31]. Therefore, the effects of microgravity on immune cells can be influenced by a range of factors, including time, gene expression, epigenomic alterations, and cellular processes. Further research will be needed to explore these variables.

In SNU-80 and BCPAP cells, the cellular response to hypoxia-related genes was downregulated. Hypoxia is a challenging tumor microenvironment with insufficient oxygen supply that cancer cells must overcome to sustain growth. In response to hypoxia, cancer cells often undergo metabolic reprogramming through the Warburg effect, bypassing the TCA cycle to survive under harsh conditions. In this process, Hypoxia-Inducible Factor 1-alpha (HIF-1α) acts as a key transcriptional regulator, modulating angiogenesis, metabolism, and cell survival. A previous study using MDA-MB-468 (human breast cancer cells) reported that gravitational changes suppress the expression of hypoxia-inducible genes caused by cytoskeleton-dependent nuclear translocation of HIFs. Macromolecules are transported and function within the cell via the cytoskeleton, but gravitational changes disrupt cytoskeletal connections. Consistently, in the present study, hypoxia-related cellular response genes were downregulated, suggesting that the reduced cellular response required for thyroid cancer cell growth could inhibit tumor progression.

In SNU-790 and TPC-1, PTC cell lines, simulated microgravity upregulated the genes associated with the cell cycle, particularly those involved in the G2-M checkpoint and mitotic spindle. The G2-M checkpoint ensures that DNA replication is complete and free of damage. If errors are detected, the cell either repairs the damage or triggers apoptosis before mitosis begins in cases of severe damage. In the present study, two key genes associated with the G2-M phase, *CDK1* and *TTK*, were upregulated. *CDK1* regulates mitotic entry by interacting with cyclins during the G2-M phase and is implicated in oncogenic pathways. *TTK*, a dual-specificity kinase, plays a role in the spindle assembly checkpoint to ensure accurate chromosome segregation during cell cycle checkpoints. Similarly, cyclin B expression increased when C2C12 mouse muscle cells were cultured in simulated microgravity, reflecting a higher proportion of cells in the G2-M phase. This resulted in delayed progression through the G2-M phase and slower proliferation. Comparable findings have been observed in human lymphocytes and breast cancer cells, where microgravity-induced cytoskeleton organization issues led to delays in the G2-M phase. Therefore, changes in the cytoskeleton may cause G2-M phase arrest in a microgravity environment, potentially inhibiting the proliferation of cancer cells.

All cell types cultured under simulated microgravity showed a tendency towards increased migration and invasion. Nevertheless, only some of these results were statistically significant, making it difficult to conclude that simulated microgravity enhances cell aggressiveness. A previous study reported increased migration in non-small cell lung cancer cells under microgravity conditions [32]. In contrast, other research observed reduced migration and invasion potential in human glioblastoma cells, indicating conflicting effects of microgravity on migration and invasion [33]. Changes in the cytoskeleton induced by microgravity can alter the cellular structures, such as the basement membrane or extracellular matrix, and may cause the cells to form 3-dimensional multicellular spheroids [4,19,32]. Although our study did not directly measure time-dependent effects, previous research suggests that cytoskeletal adaptation under microgravity may influence migration and invasion [4,34]. Since assays were conducted under normal gravity for 24 h, this transitional environment may have impacted the observed results. In addition to gene expression, factors such as cell-matrix interactions, cell-cell interactions, cytokines, mechanical forces, and cell adhesion molecules can also influence migration and invasion. Therefore, further research will be needed to clarify the conflicting findings observed in various studies and to understand the precise mechanisms through which microgravity affects cellular migration and invasion potential.

This study had certain limitations. This study implemented a simulated gravity of approximately 10e−3 g because the clinostat device cannot fully replicate true microgravity. Despite the limitation stemming from the inability to recreate microgravity conditions on Earth perfectly, experimental methods consistent with the existing literature were used to ensure that this study can serve as a valuable reference for future research in actual microgravity environments.

Additionally, this study focused only on adhesive cells. In microgravity, however, cells often grow into a 3D structure called a multicellular spheroid, which is important for replicating the tumor microenvironment because of its increased cell-to-cell interactions. Therefore, to understand the effects of microgravity, future studies should also analyze cells in the multicellular spheroid form, which can provide more realistic insights into tumor growth and drug responses.

Importantly, the findings presented here reflect correlations based on observed gene expression differences and phenotypic behaviors under different gravity conditions, and do not imply causality. While mechanotransduction, Toll-like receptor signaling, hypoxia-inducible factors, and G2–M phase regulation were proposed as potential mechanisms, this study did not directly investigate the causative roles or underlying pathways involved. Further research is required to confirm whether these pathways functionally mediate the observed changes in cellular responses under microgravity.

Furthermore, it is difficult to understand all the characteristics of cancer because this study only conducted gene expression analysis. Accordingly, additional research, such as proteomics, epigenomics, genomic instability, metabolomics, and studies on cell-to-cell interactions, will be needed to characterize cancer and validate the results of this study.

## Conclusions

This study provides new insights into the effects of simulated microgravity on various thyroid cancer cell lines, revealing distinct changes in gene expression and cellular behaviors. In particular, the cell cycle-related genes, particularly in SNU-790 and TPC-1 PTC cell lines, were upregulated. In addition, microgravity leads to the downregulation of the genes related to the HIF-1α signaling pathway in SNU-80 and BCPAP cells, while enhancing the expression of genes involved in the innate immune system in Nthy-ori 3-1, SNU-80, and BCPAP cells. Furthermore, microgravity-induced changes in gene expression could influence migration and invasion, but the precise mechanisms are unclear and appear to be time-dependent. These findings offer valuable insights into how thyroid cancer cells behave under altered gravity conditions and could have significant implications for cancer research and developing novel therapeutic strategies. Future research should aim to validate these findings, explore the underlying causal mechanisms, and assess the impact of actual microgravity on cellular behavior and other organ systems.

## Data Availability

The data that support the findings of this study are available from the corresponding author, Jin Wook Yi, upon reasonable request.

## Appendix A

**Table A1:** Differentially expressed genes (DEGs) in SNU-790 cultured under simulated microgravity compared to normal gravity. Numbers 1 to 20 represent the top 20 upregulated DEGs, and genes 21 to 40 represent the top 20 downregulated DEGs, both ranked by the absolute value of fold change

No.	Gene symbol	Fold change	Adjusted *p*-value	Gene description
1	*SNORA14A*	5.76	<0.001	Small nucleolar RNA, H/ACA box 14A
2	*HIST1H3B*	3.58	<0.001	Histone cluster 1, H3b
3	*HIST1H3G*	3.49	<0.001	Histone cluster 1, H3g
4	*TNFAIP6*	3.05	<0.001	Tumor necrosis factor, alpha-induced protein 6
5	*SNORA11*	2.99	<0.001	Small nucleolar RNA, H/ACA box 11
6	*MAGED2*	2.99	<0.001	MAGE family member D2
7	*GOLGA6L7P*	2.69	0.023	Golgin A6 family-like 7, pseudogene
8	*HIST2H2AB*	2.57	<0.001	Histone cluster 2, H2ab
9	*HIST1H1B*	2.42	<0.001	Histone cluster 1, H1b
10	*CXCL8*	2.35	<0.001	Chemokine (C-X-C motif) ligand 8
11	*METRN*	2.32	<0.001	Meteorin, glial cell differentiation regulator
12	*RNU5F-1*	2.29	<0.001	RNA, U5F small nuclear 1
13	*TUBA3C*	2.22	<0.001	Tubulin, alpha 3c
14	*SNORA38B*	2.21	<0.001	Small nucleolar RNA, H/ACA box 38B
15	*MYDGF*	2.21	<0.001	Myeloid-derived growth factor
16	*C1QTNF1*	2.21	<0.001	C1q and tumor necrosis factor related protein 1
17	*DNLZ*	2.19	0.001	DNL-type zinc finger
18	*CARD9*	2.19	0.001	Caspase recruitment domain family, member 9
19	*HIST1H2AI*	2.17	<0.001	Histone cluster 1, H2ai
20	*SNORA14A*	5.76	<0.001	Small nucleolar RNA, H/ACA box 14A
21	*MIR4524A*	−3.78	0.001	MicroRNA 4524a
22	*MIR1284*	−3.59	<0.001	MicroRNA 1284
23	*GBP3*	−3.52	0.008	Guanylate binding protein 3
24	*MIR4798*	−3.44	<0.001	MicroRNA 4798
25	*MIR4762*	−3.41	<0.001	MicroRNA 4762
26	*MIR4500*	−3.07	<0.001	MicroRNA 4500
27	*GNAI3*	−3.05	0.001	Guanine nucleotide-binding protein (G protein), alpha inhibiting activity polypeptide 3
28	*MIR4796*	−3.02	0.001	MicroRNA 4796
29	*MIRLET7A2*	−2.99	0.002	MicroRNA let-7a-2
30	*MIR1305*	−2.93	0.004	MicroRNA 1305
31	*MIR4263*	−2.92	0.002	MicroRNA 4263
32	*MIR4668*	−2.84	<0.001	MicroRNA 4668
33	*MIR548K*	−2.82	0.001	MicroRNA 548k
34	*MIR4742*	−2.80	<0.001	MicroRNA 4742
35	*IFNE*	−2.78	<0.001	Interferon, epsilon
36	*PCDHB13*	−2.78	<0.001	Protocadherin beta 13
37	*RPS15AP10*	−2.73	<0.001	Ribosomal protein S15a pseudogene 10
38	*SNORA31*	−2.69	<0.001	Small nucleolar RNA, H/ACA box 31
39	*ZNRD1-AS1*	−2.68	<0.001	ZNRD1 antisense RNA 1
40	*HCG8*	−2.68	<0.001	HLA complex group 8

**Table A2:** Differentially expressed genes (DEGs) in SNU-80 cultured under simulated microgravity compared to normal gravity. Numbers 1 to 20 represent the top 20 upregulated DEGs, and genes 21 to 40 represent the top 20 downregulated DEGs, both ranked by the absolute value of fold change

No.	Gene symbol	Fold change	Adjusted *p*-value	Gene description
1	*MIR29A*	2.08	0.001	MicroRNA 29a
2	*MIR4295*	2.03	0.015	MicroRNA 4295
3	*GBP4*	2.02	0.005	Guanylate binding protein 4
4	*MIR4668*	1.97	0.015	MicroRNA 4668
5	*IFI44L*	1.94	<0.001	Interferon-induced protein 44-like
6	*CPOX*	1.90	0.001	Coproporphyrinogen oxidase
7	*SERPINB4*	1.81	0.002	Serpin peptidase inhibitor, clade B (ovalbumin), member 4
8	*SERPINB3*	1.81	0.002	Serpin peptidase inhibitor, clade B (ovalbumin), member 3
9	*SRGAP2-AS1*	1.81	0.018	SRGAP2 antisense RNA 1
10	*TLR3*	1.80	<0.001	Toll-like receptor 3
11	*MIR507*	1.74	0.015	MicroRNA 507
12	*EVI2A*	1.73	0.009	Ecotropic viral integration site 2A
13	*MAP2K6*	1.70	0.020	Mitogen-activated protein kinase kinase 6
14	*HERC6*	1.68	0.001	HECT and RLD domain containing E3 ubiquitin protein ligase family member 6
15	*IFIT1*	1.66	0.001	Interferon-induced protein with tetratricopeptide repeats 1
16	*KLRC3*	1.66	0.011	Killer cell lectin-like receptor subfamily C, member 3
17	*NQO1*	1.65	0.006	NAD(P)H dehydrogenase, quinone 1
18	*GBP1*	1.62	0.001	Guanylate binding protein 1, interferon-inducible
19	*IFIH1*	1.62	0.001	Interferon induced, with helicase C domain 1
20	*TLR6*	1.62	0.020	Toll-like receptor 6; toll-like receptor 1
21	*CA9*	−3.70	0.035	Carbonic anhydrase IX
22	*NRN1*	−3.19	0.036	Neuritin 1
23	*PDK1*	−2.52	0.038	Pyruvate dehydrogenase kinase, isozyme 1
24	*NPPB*	−2.44	0.001	Natriuretic peptide B
25	*TMEM45A*	−2.26	0.007	Transmembrane protein 45A
26	*EDN1*	−2.24	<0.001	Endothelin 1
27	*VLDLR*	−2.21	0.004	Very low-density lipoprotein receptor
28	*OR10V2P*	−2.15	<0.001	Olfactory receptor, family 10, subfamily V, member 2 pseudogene
29	*NDRG1*	−2.15	0.019	N-myc downstream regulated 1
30	*LOX*	−2.12	<0.001	Lysyl oxidase
31	*COL11A1*	−2.08	0.019	Collagen, type XI, alpha 1
32	*MIR320D1*	−2.04	0.001	MicroRNA 320d-1
33	*ENO2*	−2.03	0.022	Enolase 2 (gamma, neuronal)
34	*TCAF2*	−1.90	0.015	TRPM8 channel-associated factor 2
35	*LIMCH1*	−1.87	<0.001	LIM and calponin homology domains 1
36	*KCTD11*	−1.87	0.043	Potassium channel tetramerization domain containing 11
37	*MIR210HG*	−1.87	0.044	MIR210 host gene
38	*UPK1A*	−1.83	0.002	Uroplakin 1A
39	*STC1*	−1.83	0.015	Stanniocalcin 1
40	*HK2*	−1.81	0.008	Hexokinase 2

**Table A3:** Differentially expressed genes (DEGs) in Nthy-ori 3-1 cultured under simulated microgravity compared to normal gravity. Numbers 1 to 20 represent the top 20 upregulated DEGs, and genes 21 to 40 represent the top 20 downregulated DEGs, both ranked by the absolute value of fold change

No.	Gene symbol	Fold change	Adjusted *p*-value	Gene description
1	*GBP1*	2.43	<0.001	Guanylate binding protein 1, interferon-inducible
2	*FOSB*	2.37	<0.001	FBJ murine osteosarcoma viral oncogene homolog B
3	*DUSP5*	2.11	<0.001	Dual specificity phosphatase 5
4	*MIR3135B*	1.91	0.012	MicroRNA 3135b
5	*KCNJ2*	1.90	<0.001	Potassium channel, inwardly rectifying subfamily J, member 2
6	*PTGER4*	1.90	<0.001	Prostaglandin E receptor 4 (subtype EP4)
7	*MAP1LC3C*	1.88	0.001	Microtubule associated protein 1 light chain 3 gamma
8	*MT1G*	1.87	0.040	Metallothionein 1G
9	*SULT1E1*	1.86	<0.001	Sulfotransferase family 1E member 1
10	*SULT1D1P*	1.86	<0.001	Sulfotransferase family 1D member 1, pseudogene
11	*MIR4739*	1.85	0.031	MicroRNA 4739
12	*MIR1471*	1.79	0.034	MicroRNA 1471
13	*ANXA1*	1.78	0.001	Annexin A1
14	*MRS2P2*	1.78	0.031	MRS2 pseudogene 2
15	*IFIT2*	1.78	<0.001	Interferon-induced protein with tetratricopeptide repeats 2
16	*TLR3*	1.76	<0.001	Toll-like receptor 3
17	*MIR1271*	1.74	0.001	MicroRNA 1271
18	*IFI30*	1.74	<0.001	Interferon, gamma-inducible protein 30
19	*MIR554*	1.73	<0.001	MicroRNA 554
20	*MIR519E*	1.73	0.004	MicroRNA 519e
21	*FER1L4*	−3.10	<0.001	Fer-1-like family member 4, pseudogene (functional)
22	*LUCAT1*	−2.79	<0.001	Lung cancer associated transcript 1 (non-protein coding)
23	*MIR3617*	−2.48	0.001	MicroRNA 3617
24	*CA9*	−2.47	<0.001	Carbonic anhydrase IX
25	*LINC01583*	−2.44	<0.001	Long intergenic non-protein coding RNA 1583
26	*NDRG1*	−2.43	<0.001	N-myc downstream regulated 1
27	*MIR374A*	−2.33	0.001	MicroRNA 374a
28	*BNIP3*	−2.31	<0.001	BCL2/adenovirus E1B 19kDa interacting protein 3
29	*SPAG4*	−2.21	<0.001	Sperm associated antigen 4
30	*INHBB*	−2.17	<0.001	Inhibin beta B
31	*ADAM12*	−2.12	<0.001	ADAM metallopeptidase domain 12
32	*STC1*	−2.07	<0.001	Stanniocalcin 1
33	*LOX*	−2.02	<0.001	Lysyl oxidase
34	*IGFBP3*	−1.97	<0.001	Insulin like growth factor binding protein 3
35	*KCNMA1*	−1.97	<0.001	Potassium channel, calcium activated large conductance subfamily M alpha, member 1
36	*VEGFA*	−1.89	<0.001	Vascular endothelial growth factor A
37	*PAG1*	−1.87	<0.001	Phosphoprotein membrane anchor with glycosphingolipid microdomains 1
38	*CDON*	−1.87	0.002	Cell adhesion associated, oncogene regulated
39	*F3*	−1.87	<0.001	Coagulation factor III (thromboplastin, tissue factor)
40	*SPINT4*	−1.86	<0.001	Serine peptidase inhibitor, Kunitz type 4

**Table A4:** Differentially expressed genes (DEGs) in TPC-1 cultured under simulated microgravity compared to normal gravity. Numbers 1 to 20 represent the top 20 upregulated DEGs, and genes 21 to 40 represent the top 20 downregulated DEGs, both ranked by the absolute value of fold change

No.	Gene symbol	Fold change	Adjusted *p*-value	Gene description
1	*FOSB*	5.54	<0.001	FBJ murine osteosarcoma viral oncogene homolog B
2	*ATF3*	2.91	<0.001	Activating transcription factor 3
3	*MIR323A*	2.46	0.020	MicroRNA 323a
4	*MIR27A*	2.40	<0.001	MicroRNA 27a
5	*FOS*	2.33	<0.001	FBJ murine osteosarcoma viral oncogene homolog
6	*MIR23A*	2.27	<0.001	MicroRNA 23a
7	*VTRNA1-1*	2.24	0.009	Vault RNA 1-1
8	*KCNJ6*	2.23	<0.001	Potassium channel, inwardly rectifying subfamily J, member 6
9	*MIR24-2*	2.19	<0.001	MicroRNA 24-2
10	*HES1*	2.17	<0.001	Hes family bHLH transcription factor 1
11	*MIR222*	2.13	<0.001	MicroRNA 222
12	*HIST1H3G*	2.13	<0.001	Histone cluster 1, H3g
13	*HIST1H2AI*	2.07	<0.001	Histone cluster 1, H2ai
14	*SPANXC*	2.05	0.032	SPANX family, member C
15	*CXCL8*	2.04	0.002	Chemokine (C-X-C motif) ligand 8
16	*LDLR*	2.03	<0.001	Low density lipoprotein receptor
17	*DUSP5*	1.98	<0.001	Dual specificity phosphatase 5
18	*HIST1H2BB*	1.95	<0.001	Histone cluster 1, H2bb
19	*HIST1H2BM*	1.95	0.001	Histone cluster 1, H2bm
20	*HIST2H2AB*	1.91	<0.001	Histone cluster 2, H2ab
21	*MIR548T*	−3.56	<0.001	MicroRNA 548t
22	*MIR548AM*	−3.23	0.015	MicroRNA 548am
23	*CHAC1*	−2.55	<0.001	ChaC glutathione-specific gamma-glutamylcyclotransferase 1
24	*LINC01111*	−2.50	<0.001	Long intergenic non-protein coding RNA 1111
25	*LINC01109*	−2.50	<0.001	Long intergenic non-protein coding RNA 1109
26	*MIR507*	−2.48	<0.001	MicroRNA 507
27	*SLC7A11*	−2.42	<0.001	Solute carrier family 7 (anionic amino acid transporter light chain, xc- system), member 11
28	*ZPLD1*	−2.41	<0.001	Zona pellucida-like domain containing 1
29	*ACSM2A*	−2.13	<0.001	Acyl-CoA synthetase medium-chain family member 2A
30	*MIR23B*	−2.06	<0.001	MicroRNA 23b
31	*C9orf3*	−2.06	<0.001	Chromosome 9 open reading frame 3
32	*MIR4791*	−2.04	0.018	MicroRNA 4791
33	*MIR153-2*	−2.02	0.025	MicroRNA 153-2
34	*DEFB115*	−1.99	0.002	Defensin, beta 115
35	*AMELX*	−1.93	0.007	Amelogenin, X-linked
36	*MIR181B2*	−1.92	<0.001	MicroRNA 181b-2
37	*TSPAN2*	−1.91	<0.001	Tetraspanin 2
38	*PSG1*	−1.89	0.002	Pregnancy specific beta-1-glycoprotein 1
39	*SMPDL3A*	−1.88	<0.001	Sphingomyelin phosphodiesterase, acid-like 3A
40	*MIR181B1*	−1.86	<0.001	MicroRNA 181b-1

**Table A5:** Differentially expressed genes (DEGs) in BCPAP cultured under simulated microgravity compared to normal gravity. Numbers 1 to 20 represent the top 20 upregulated DEGs, and genes 21 to 40 represent the top 20 downregulated DEGs, both ranked by the absolute value of fold change

No.	Gene symbol	Fold change	Adjusted *p*-value	Gene description
1	*GBP4*	6.24	<0.001	Guanylate binding protein 4
2	*CXCL10*	5.39	<0.001	Chemokine (C-X-C motif) ligand 10
3	*MMP3*	5.33	<0.001	Matrix metallopeptidase 3
4	*IL1B*	5.03	<0.001	Interleukin 1 beta
5	*MMP13*	4.99	<0.001	Matrix metallopeptidase 13
6	*IFIT2*	4.02	<0.001	Interferon-induced protein with tetratricopeptide repeats 2
7	*C15orf48*	3.99	<0.001	Chromosome 15 open reading frame 48
8	*MIR147B*	3.99	<0.001	MicroRNA 147b
9	*VCAM1*	3.98	<0.001	Vascular cell adhesion molecule 1
10	*MMP12*	3.98	<0.001	Matrix metallopeptidase 12
11	*MIR3142*	3.69	<0.001	MicroRNA 3142
12	*IFIT3*	3.62	<0.001	Interferon-induced protein with tetratricopeptide repeats 3
13	*GBP5*	3.62	<0.001	Guanylate binding protein 5
14	*TNFSF10*	3.27	<0.001	Tumor necrosis factor (ligand) superfamily, member 10
15	*IFIT1*	3.20	<0.001	Interferon-induced protein with tetratricopeptide repeats 1
16	*HMGN2P46*	3.09	<0.001	High mobility group nucleosomal binding domain 2 pseudogene 46
17	*SOD2*	3.07	<0.001	Superoxide dismutase 2, mitochondrial
18	*CMPK2*	3.07	<0.001	Cytidine monophosphate (UMP-CMP) kinase 2, mitochondrial
19	*IDO1*	3.02	<0.001	Indoleamine 2,3-dioxygenase 1
20	*CXCL11*	3.02	<0.001	Chemokine (C-X-C motif) ligand 11
21	*CA9*	−7.22	<0.001	Carbonic anhydrase IX
22	*BNIP3*	−5.59	<0.001	BCL2/adenovirus E1B 19kDa interacting protein 3
23	*SNORD114-31*	−4.78	<0.001	Small nucleolar RNA, C/D box 114-31
24	*MIR136*	−4.72	<0.001	MicroRNA 136
25	*PTPRB*	−4.32	<0.001	Protein tyrosine phosphatase, receptor type, B
26	*LOX*	−4.15	<0.001	Lysyl oxidase
27	*MIR431*	−3.99	<0.001	MicroRNA 431
28	*MIR432*	−3.59	<0.001	MicroRNA 432
29	*C4orf47*	−3.36	<0.001	Chromosome 4 open reading frame 47
30	*MIR433*	−3.31	<0.001	MicroRNA 433
31	*RTL1*	−3.30	<0.001	Retrotransposon-like 1
32	*MIR3617*	−3.23	<0.001	MicroRNA 3617
33	*FER1L4*	−3.20	<0.001	Fer-1-like family member 4, pseudogene (functional)
34	*MIR127*	−3.16	<0.001	MicroRNA 127
35	*PDK1*	−3.10	<0.001	Pyruvate dehydrogenase kinase, isozyme 1
36	*SNORD113-1*	−2.76	<0.001	Small nucleolar RNA, C/D box 113-1
37	*SNORD114-16*	−2.66	<0.001	Small nucleolar RNA, C/D box 114-16
38	*ENO2*	−2.53	<0.001	Enolase 2 (gamma, neuronal)
39	*CITED2*	−2.49	<0.001	Cbp/p300-interacting transactivator, with Glu/Asp rich carboxy-terminal domain, 2
40	*SH3D21*	−2.46	<0.001	SH3 domain containing 21
